# Mesencephalic astrocyte-derived neurotropic factor is an important factor in chondrocyte ER homeostasis

**DOI:** 10.1007/s12192-018-0953-7

**Published:** 2018-12-12

**Authors:** P. A. Bell, E. P. Dennis, C. L. Hartley, R. M. Jackson, A. Porter, R. P. Boot-Handford, K. A. Pirog, M. D. Briggs

**Affiliations:** 1 Institute of Genetic Medicine, International Centre for Life, Newcastle University, Newcastle Upon Tyne, NE1 3BZ UK; 2 Present Address: Centre for Blood Research, University of British Columbia, Vancouver, BC V6T 1Z3 Canada; 3 Wellcome Trust Centre for Cell-Matrix Research, University of Manchester, Oxford Road, Manchester, M13 9PT UK; 4 Present Address: Genomic Diagnostics Laboratory, Manchester Centre for Genomic Medicine, Central Manchester University Hospitals NHS Foundation Trust, Manchester, M13 9WL UK; 5 Newcastle University Protein and Proteome Analysis Facility, Newcastle University, Newcastle Upon Tyne, NE1 7RU UK

**Keywords:** UPR, MANF, Skeletal development, Chondrodysplasia

## Abstract

Mesencephalic astrocyte-derived neurotrophic factor (MANF) is an endoplasmic reticulum (ER) resident protein that can be secreted due to an imperfect KDEL motif. MANF plays a cytoprotective role in several soft tissues and is upregulated in conditions resulting from intracellular retention of mutant protein, including two skeletal diseases, metaphyseal chondrodysplasia, Schmid type (MCDS) and multiple epiphyseal dysplasia (MED). The role of MANF in skeletal tissue homeostasis is currently unknown. Interestingly, cartilage-specific deletion of *Manf* in a mouse model of MED resulted in increased disease severity, suggesting its upregulation may be chondroprotective. Treatment of MED chondrocytes with exogenous MANF led to a decrease in the cellular levels of BiP (GRP78), confirming MANF’s potential to modulate ER stress responses. However, it did not alleviate the intracellular retention of mutant matrilin-3, suggesting that it is the intracellular MANF that is of importance in the pathobiology of skeletal dysplasias. The *Col2Cre*-driven deletion of *Manf* from mouse cartilage resulted in a chondrodysplasia-like phenotype. Interestingly, ablation of MANF in cartilage did not have extracellular consequences but led to an upregulation of several ER-resident chaperones including BiP. This apparent induction of ER stress in turn led to dysregulated chondrocyte apoptosis and decreased proliferation, resulting in reduced long bone growth. We have previously shown that ER stress is an underlying disease mechanism for several skeletal dysplasias. The cartilage-specific deletion of *Manf* described in this study phenocopies our previously published chondrodysplasia models, further confirming that ER stress itself is sufficient to disrupt skeletal growth and thus represents a potential therapeutic target.

**Electronic supplementary material:**

The online version of this article (10.1007/s12192-018-0953-7) contains supplementary material, which is available to authorized users.

## Introduction

Mesencephalic astrocyte-derived neurotrophic factor (MANF) is an endoplasmic reticulum (ER)-stress associated protein that was initially identified as a neuroprotective factor in dopaminergic neurons (Hellman et al. 2011; Petrova et al. 2003). MANF has neuroprotective and cardioprotective effects, especially against ischemia-induced ER stress, and can promote cell proliferation in several tissues (Airavaara et al. 2009; Apostolou et al. 2008; Tadimalla et al. 2008). Mutations in *MANF* have been found in several tumours (Tanaka et al. 2000; Shridhar et al. 1997; Shridhar et al. 1996a, b), whilst increased expression of MANF has also been demonstrated in cytokine-induced ER stress (Cunha et al. 2017) and in diseases where the unfolded protein response (UPR) is induced by a misfolded mutant protein, such as in metaphyseal chondrodysplasia, Schmid type (MCDS; OMIM #156500 and resulting from mutations in *COL10A1*) or multiple epiphyseal dysplasia (MED; #607078 and resulting from mutations in *MATN3*) (Leighton et al. 2007; Nundlall et al. 2010; Cameron et al. 2011).

MANF is an ER-resident protein; however, it contains an imperfect KDEL sequence that allows it to be secreted under ER stress, suggesting it could be used as a biomarker for a subset of ER stress-related conditions (Henderson et al. 2013; Oh-Hashi et al. 2012). Moreover, exogenous MANF has been shown to have cytoprotective effects, potentially exerted via cell surface KDEL receptors (Henderson et al. 2013), and works in conjunction with the canonical ER chaperone BiP (GRP78). However, data on the effect of exogenous MANF on BiP expression suggests that this relationship might be cell or stress-type specific (Apostolou et al. 2008; Huang et al. 2016; Zhao et al. 2013).

*Manf* was identified as one of the most highly upregulated genes in the transcriptomic analysis of a mouse model of MED resulting from a mutation in the gene encoding matrilin-3, a structural molecule of the cartilage extracellular matrix (ECM) (Nundlall et al. 2010). *MATN3*-related MED results from canonical ER stress induced by the expression and misfolding of mutant matrilin-3 that aggregates over time in the ER lumen (Leighton et al. 2007; Nundlall et al. 2010). *Manf* was also upregulated in a related skeletal dysplasia, MCDS, which results from misfolding of type X collagen, a cartilage ECM protein exclusively expressed by hypertrophic chondrocytes of the growth plate (Hartley et al. 2013; Cameron et al. 2011).

MANF protein possesses several CXXC motifs that are shared amongst protein disulphide isomerases, suggesting a potential role in protein folding; however, we have previously shown that although MANF is present in the ER, it does not function as a protein disulphide isomerase (PDI) (Hartley et al. 2013).

The role of MANF in regulating the UPR, specifically during development of unchallenged healthy tissues, remains largely unknown. In this paper, we present data showing the importance of MANF in cartilage development and show that MANF exists in the chondrocyte ER in an interaction complex with several other ER resident chaperone proteins. Ablation of MANF from cartilage led to an imbalance of the UPR machinery and induced a protein kinase R (PKR)-like ER kinase (PERK)-mediated ER stress response that in turn resulted in decreased chondrocyte proliferation and reduced long bone growth. The results of this study further confirm that the induction of ER stress, whether by the expression of a mutant protein, or a genetically engineered deregulation of the UPR, results in a chondrodysplasia-like phenotype.

## Methods

### Generation of transgenic animals

*Manf* null mice were generated using targeted C57BL6 mouse embryonic stem cell clone *Manf*^*tm1a(KOMP)Wtsi*^ obtained from the KOMP Repository (http://www.komp.org). All the mice were generated on the C57BL6/J background to control for the genetic background effects. C57BL6 blastocysts were provided by the Animal Unit at University of Manchester, resulting in C57BL6 pure *Manf* null line (*Manf*^−/−^). Southern blotting was performed to confirm correct targeting (not shown), and Western blotting was performed on cartilage to confirm deletion of MANF in mutant chondrocytes. *Manf* conditional line (*Manf*^*fl/fl*^ *Col2Cre*^+^) was generated by restoring the wild-type allele through crossing the *Manf*^−/−^ mice with an *Actin-Flp* (C57BL6) mouse and then by crossing the mice with a C57BL6 *Col2Cre* expressing line (Sakai et al. 2001). To analyse the expression pattern of *Col2cre*, mice expressing the *Col2Cre* transgene were crossed with mice homozygous for the *Gtrosa26*^*tm1Sor*^-targeted mutation consisting of a bacterial β-galactosidase gene flanked by two loxP sites. All experiments were performed in compliance with the Scientific Procedures Act of 1986 and the relevant Home Office (under PPL 40/2884 and PPL60/04525) and Institutional regulations governing animal breeding and handling.

### LacZ staining of embryos and tissues

*Gtrosa26*^*tm1Sor*^ mice were bred with a Cre expressing strain, which resulted in removal of a DNA fragment that prevents transcription of the lacZ gene in the tissues expressing Cre recombinase. Tissue expression pattern of the Cre transgene was then assayed using X-gal staining. *Gtrosa26*^*tm1Sor*^;*Col2Cre*^+^ and *Gtrosa26*^*tm1Sor*^;*Col2Cre*^−^ embryos at embryonic day 12.5 were fixed for 15 min in 2.0% formaldehyde and 0.2% glutaraldehyde. Postnatal 1-week old limbs from *Gtrosa26*^*tm1Sor*^;*Col2Cre*^+^ and *Gtrosa26*^*tm1Sor*^;*Col2Cre*^−^ mice were harvested and fixed in 10% neutral buffered formalin for 4 h at room temperature. The samples were washed in 0.01% sodium deoxycholate and 0.02% Nonidet™ P-40 and placed in lacZ staining solution containing 1 mg/ml X-Gal overnight in a humidified darkened chamber at 37 °C. Following a wash in 1× PBS, the embryos were fixed in 10% neutral buffered formalin overnight and incubated in 1% formaldehyde solution for long-term storage. The limbs were decalcified by gentle agitation in 20% EDTA pH 7.4, embedded in OCT medium and sectioned at 10-μm sections using Leica CM1860 Cryostat. Sections were counterstained with eosin, dehydrated through a series of ethanol concentrations and mounted in HistoMount™.

### Bone measurements

X-rays were acquired using the MX-20 Cabinet X-ray System (Faxitron® Bioptics) under transient anaesthesia, allowing for longitudinal bone growth measurements at 3 and 9 weeks of age. Bones were measured using ImageJ (National Institutes of Health, Bethesda, MD, USA; Rueden et al. 2017). One-way ANOVA was performed using GraphPad Prism version 7.0 (GraphPad Software, La Jolla, CA, USA, www.graphpad.com) for statistical analysis of the data.

### Histology and immunohistochemistry

Embryos were retrieved by caesarean section at E18.5, and their lungs were collected. One-week-old mice and adult mice were culled by anaesthetic overdose, and hind limbs were harvested. Hind limbs and lungs were fixed in either 4% paraformaldehyde (PFA, for histology) or 95% ethanol 5% acetic acid (immunohistochemistry) for 48 h at 4 °C. The limbs were then decalcified in 20% EDTA pH 7.4 for 2 weeks, embedded in paraffin and cut into 6-μm sections. Haematoxylin/eosin (H&E) staining was performed to visualise the general morphology of the tissue, using the Thermo Scientific™ Linistat™ Slide Stainer. To analyse the expression pattern of the *Col2cre* transgene in the limbs, 1-week-old PFA-fixed sections were stained overnight in a LacZ staining solution containing 1 mg/mL X-Gal. Immunohistochemistry and bromodeoxyuridine (BrdU) labelling were performed as described previously (Pirog-Garcia et al. 2007) using the appropriate Alexa Fluor® secondary antibodies. For immunohistochemistry, slides were mounted in Fluoroshield™ Mounting Media with DAPI (Abcam®). Primary antibodies were used at a dilution of 1:500 (type II collagen (ab34712, Abcam®); Matrilin-3 (AF3357, R&D Systems®; type X collagen); Rajpar et al. 2009). Images were obtained using the Zeiss Axio Imager 2 microscope. BrdU-labelled cells were counted using the watershed algorithm on the Fiji ImageJ platform (National Institutes of Health, Bethesda, MD, USA; Schindelin et al. 2012) and presented as percentage of total cells in the proliferative zone.

### TUNEL assay

TUNEL assay was used to analyse cell death on 4% PFA fixed sections of 3-week-old limbs using the Promega® DeadEnd™ Fluorometric TUNEL System according to the manufacturer’s protocol. Antigen unmasking was performed using a citric buffer boil pH 6 boil instead of proteinase K unmasking that can generate false positives (Gál et al. 2000; Pirog-Garcia et al. 2007). Sections were mounted in Fluoroshield™ mounting media with DAPI, and images were obtained using the Zeiss Axio Imager 2 microscope. Positive TUNEL cells were counted using the watershed algorithm on Fiji ImageJ platform (National Institutes of Health, Bethesda, MD, USA; Schindelin et al. 2012) and presented as percentage of all cells in selected zones of the growth plate. One-way ANOVA was performed using GraphPad Prism version 7.0 (GraphPad Software, La Jolla CA, USA, www.graphpad.com) for statistical analysis of the data.

### RNA extraction and RNA-sequencing analysis of knee chondrocytes

Whole knee joints from 5-day-old mice were dissected and cleaned free of soft tissue following digestion for 1 h in 2.5 mg/mL collagenase IA. Joints were homogenised using a Satorius Mikro-Dismembranator S, and RNA was extracted using the Promega® ReliaPrep™ RNA Tissue Miniprep System according to manufacturer’s protocol.

The extracted RNA was sent to GATC Biotech for analysis. Each sample contained RNA extracted from three littermates, pooled into one sample at a concentration of 1 μg total RNA with a RNA integrity number (RIN) ≥ 8. The RNA was sequenced and analysed by GATC Biotech. Briefly, RNA sequencing reads were aligned to the mouse reference genome using Bowtie. Top Hat was used to identify exon–exon splice junctions of the initial alignment. Cufflinks and Cuffmerge were used to identify, quantify, merge and annotate the transcripts from the processed RNA-Seq alignment assembly. The merged transcripts from the wild type (*Manf*^*fl/fl*^ *Col2Cre*^−^) and *Manf*^*fl/fl*^ *Col2Cre*^+^ samples were compared using Cuffdiff to determine differential expression levels with a measure of significance between the samples.

### Extraction and treatment of primary chondrocytes

Costal and tibial chondrocytes were isolated from pooled litters of 5-day-old mice (Nundlall et al. 2010) and cultured for up to a week in DMEM/F12 GlutaMAX™ supplement medium (Fisher Scientific™) containing 5% FBS, 5% non-essential amino acids, 1 U/mL penicillin, 1 μg/mL streptomycin and 50 g/mL l-ascorbate-2-phosphate.

Once confluent, the extracted cells were treated with 1 mM thapsigargin or 1 μg/mL tunicamycin in DMSO to induce ER stress. After 4 h, 100 ng/mL recombinant human MANF (3748-MN-050, R&D Systems®) was added to the media and cells were cultured for a further 24 h. To analyse the effect of MANF on primary V194D matrilin-3 chondrocytes, 100 ng/mL recombinant human MANF was added to media and cells were cultured for 24 h.

### Protein extraction and immunoblotting

Protein lysates were prepared from cells in monolayer in 1× RIPA buffer (50 mM Tris–HCl, pH 7.4, 1% Triton X-100, 150 mM NaCl, 1 mM EDTA) and centrifugation at 13,600×*g*. Samples were denatured by boiling at 95 °C for 5 min in SDS-PAGE loading buffer containing 100 mM dithiothreitol (DTT) for reducing conditions. Proteins were separated according to size by SDS-PAGE using precast Novex™ NuPAGE® 4–12% Bis-Tris precast gels (Fisher Scientific™) in MES SDS-PAGE running buffer (Fisher Scientific™) at 200 V for 60 min. Proteins were then electroblotted onto a nitrocellulose membrane for 1 h at 30 V using the XCell II™ Blot Module (Fisher Scientific™). Gel loading was assessed using REVERT™ Total Protein Stain (LI-COR® Biosciences). The membrane was then destained using REVERT™ Reversal Solution according to manufacturer’s instructions. Membranes were washed and blocked in 3% milk in PBS-T then incubated with primary antibodies for 1 h at room temperature. Primary antibodies used were GRP78 (ab108615, Abcam®), GRP94 (sc-1794, Santa Cruz Biotechnology), PDIA6 (ab154820, Abcam®) and matrilin-3 (AF3357, R&D). Membranes were then probed with the appropriate LI-COR® IRDye® secondary antibody at a concentration of 1:10,000 for 1 h. Blots were imaged on the LI-COR® Odyssey CLx Imaging System, and band intensity was normalised to the total protein stain using the LI-COR® proprietary software. Loading control images (total protein) are included in Online Resource 1, Fig. 3. Student’s *t* test was performed using GraphPad Prism version 7.0 (GraphPad Software, La Jolla, CA, USA, www.graphpad.com) for statistical analysis of the data.

### Co-immunoprecipitation

Co-immunoprecipitation (Co-IP) was carried out on cell lysates from HEK-293 cells transfected with FLAG-tagged and V5-tagged wild-type MANF constructs in pcDNA3.1+ using anti-FLAG and anti-V5 agarose beads (ab1299, Abcam®). Prior to immunoprecipitation, cells were treated with 1 mM dithiobis[succinimidyl propionate] (DSP) to stabilise protein complexes as previously described (Cotterill et al. 2005). Cell lysates were prepared as outlined above. Co-IP was performed using 500 μg of cell lysate, which was incubated with anti-V5 agarose beads overnight under gentle agitation as per manufacturer’s instructions. The resin was pelleted by centrifugation and washed three times in 1× RIPA buffer. Proteins were eluted in SDS buffer, and SDS-PAGE was performed as above.

### Mass spectrometry and data analysis

Twenty-microliter aliquots of cross-linked Co-IP cell-lysates were run into 4–12% SDS-polyacrylamide gels for 4 min (at 200 V). Total protein pools were excised from the gel, dehydrated, reduced, alkylated and washed. Samples were then digested with trypsin overnight at 37 °C and analysed by LC-MS/MS. Peptides were concentrated on a precolumn (C18 PepMap, 20 mm × 180-μm i.d, Thermo) and separated using a gradient from 95% A (0.1% formic acid in 2% acetonitrile) and 5% B (0.1% formic acid in acetonitrile) to 35% B, in 132 min at 300 nL min^−1^, using a 75-μm × 250-mm i.d. 3-μm particle size PepMap C18, analytical column (Waters) on an Ultimate 3000 nano-HPLC system (Thermo) coupled to an LTQ Orbitrap XL mass spectrometer (Thermo). The capillary voltage was set to 200 °C and the spray voltage to 1.6 kV. Survey scans were acquired in the Orbitrap with a resolution of 30,000 at *m*/*z* 400 Da. Up to 15 data-dependent MSMS scans were acquired in the LTQ following CID. Peak lists were generated using msconvert, and the data was searched using X!Tandem and the GPM interface against a concatenation of the ENSEMBL *Homo sapiens* genome (v. 76) and cRAP (v.2012.01.01) (Kessner et al. 2008; Craig et al. 2004). The following search parameters were used: tryptic cleavage; precursor mass accuracy 100 ppm; product ion mass tolerance 0.6 Da; static modification- carbamidomethylation on C; variable modification- oxidation on Met; refinement- yes; refinement modifications- Deamidated@N or Q, Phospho@S or T or Y, Oxidation@M or W, Methyl@C or D or E or H or R or K or N or Q, Dioxidation@M or W, Dehydrated@S or T, -Carbamidomethyl@C, Carbamidomethyl@H or D or E or K. Proteins were considered to be identified when they had protein level log(e) values < (− 3). Proteins present in cRAP were removed from the result lists. Three biological replicates were used in all experiments. The number of spectra identified for each protein was compared between wild-type and mutant genotypes using the Student’s *t* test GraphPad Software, La Jolla, CA, USA, www.graphpad.com) with a *P* value < 0.05 was considered significant.

## Results

### Global deletion of Manf leads to perinatal lethality due to breathing difficulties induced by lung malformation

**Fig. 1:**
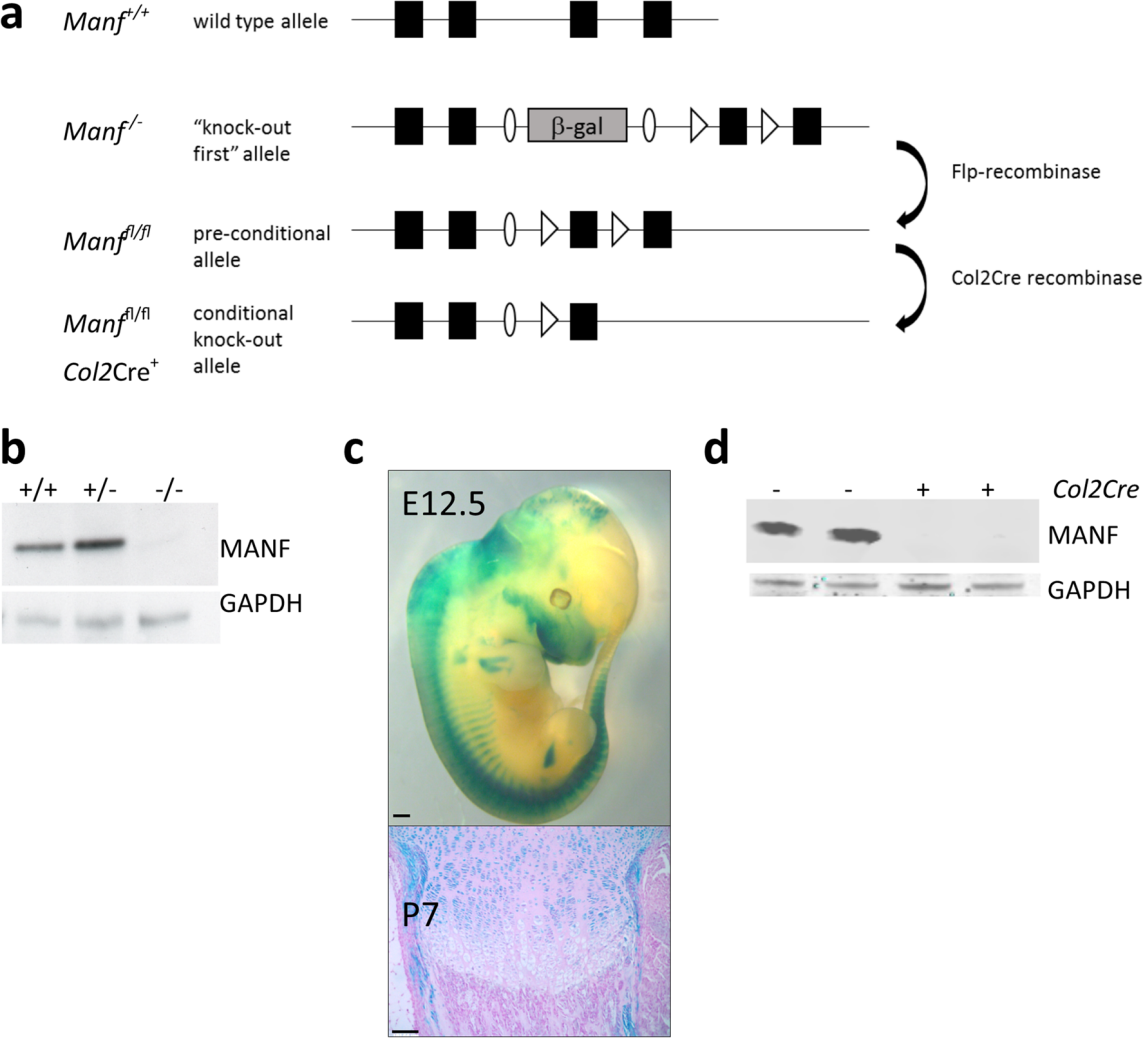
**a** Targeting strategy used to generate the *Manf*^−/−^ (‘knockout first’) and *Manf*^fl/fl^ *Col2Cre*^+^ (conditional knockout allele) lines. Black boxes represent *Manf* exons; LoxP sites are marked by arrowheads and FRT sites by ovals. **b** Western blotting showing the deletion of MANF in *Manf*^−/−^ liver at E19.5, GAPDH as loading control. **c** LacZ staining of the whole mount ß-galactosidase (Rosa26) reporter crossed with *Col2Cre* line embryo at E15.5, showing cartilage-specific expression of the *Col2Cre* transgene. **d** Western blotting showing deletion of MANF in *Manf*^fl/fl^ *Col2Cre*^+^ cartilage at P21, GAPDH as loading control. Scale bars 1 mm and 100 μm

*Manf* null mice were generated using targeted C57BL/6 mouse embryonic stem cell clones obtained from the KOMP Repository (http://www.komp.org,). The clones were injected into C57BL/6 blastocysts resulting in a *Manf* null line (*Manf*^−/−^; Fig. 1a). The lack of MANF expression was confirmed by Western blotting in liver tissue samples (Fig. 1b). Interestingly, heterozygous breeding pairs (*Manf*^+/−^ × *Manf*^+/−^) failed to produce *Manf* null offspring, with a genotype ratio of *Manf*^+/−^:*Manf*^+/−^:*Manf*^−/−^ offspring of 33%:67%:0% over 14 litters from 3 different breeding pairs. Embryos were therefore recovered at E18.5 by caesarean section in an attempt to foster; however, they failed to breathe when induced.

In order to understand the underlying pathology of these mice, the lungs were collected from embryos at E17.5, E18.5 and E19.5 days to analyse the respiratory capacity of the animals and to identify any potential respiratory impairment. Following haematoxylin and eosin of lung sections, the alveolar volume was measured using ImageJ. The alveolar volume was significantly decreased in the *Manf* null mice at all ages analysed, thus confirming a role for *Manf* in embryonic lung development (Online Resource 1, Fig. 1a, b).

### Endochondral ossification is impaired in cartilage-specific Manf knockout mice

Based on our previous studies (Piróg et al. 2014), and other published evidence (Dixon and Dixon 2004; McLaughlin et al. 2007), we have hypothesised that a C57BL/6 genetic background can exaggerate the skeletal disease phenotype in ER stress-related mouse models. Indeed, crossing the global *Manf* null mice onto a 129Sv background (50%:50% C57BL/6:129Sv) resulted in viable offspring, which is consistent with a previously published study (Lindahl et al. 2014). However, global *Manf* null mice were much smaller than their littermates and later became diabetic (Online Resource 1, Fig. 2a). In order to eliminate systemic and metabolic effects on bone growth, and to study the specific role of *Manf* in cartilage development and homeostasis, we generated a conditional knockout mouse line. *Manf* null animals were crossed with a *Flp* recombinase strain to restore a wild-type allele and then with a *Col2Cre* expressing mouse (Gualeni et al. 2013) (Fig. 1c) in order to delete exon 3 of *Manf* in cartilage only (Fig. 1a). SDS-PAGE and Western blotting were performed on femoral head cartilage dissected from 5-day-old mice to confirm that MANF was absent in *Manf*^*fl/fl*^ *Col2Cre*^+^ mice (Fig. 1d).

**Fig. 2:**
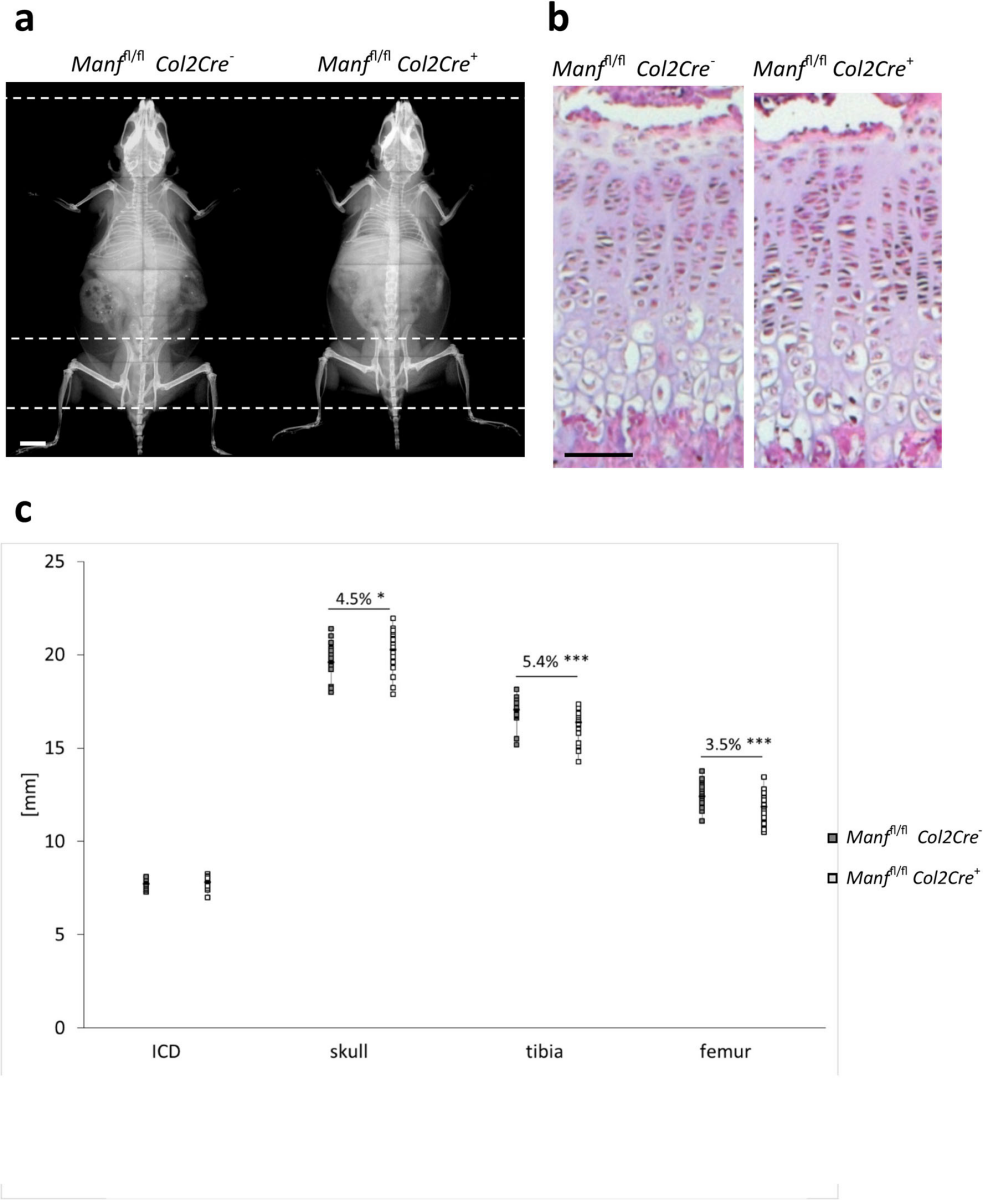
X-ray radiographs of *Manf*^fl/fl^ *Col2Cre*^−^ and *Col2Cre*^+^ mice at 9 weeks. **b** Haematoxylin and eosin staining of *Manf*^fl/fl^ *Col2Cre*^−^ and *Col2Cre*^+^ cartilage growth plates at P21. **c** Bone measurements of *Manf*^fl/fl^ *Col2Cre*^−^ and *Col2Cre*^+^ mice at P63 showing affected endochondral ossification elements (*n* > 5). RZ resting zone, PZ proliferative zone, HZ hypertrophic zone. *P < 0.05, ***P < 0.0001. Scale bars 5 mm (**a**) and 200 μm (**b**)

**Fig. 3:**
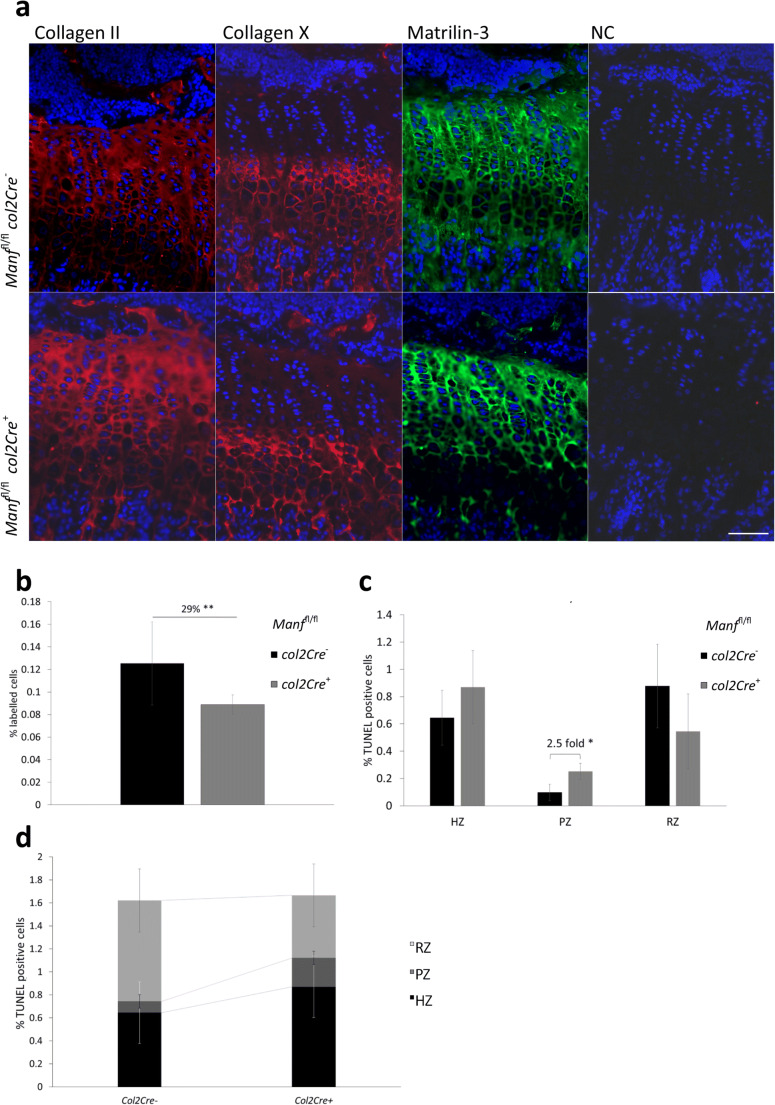
**a** Immunohistochemistry showing the localisation of cartilage extracellular matrix components, type II collagen, type X collagen and matrilin-3 in *Manf*^fl/fl^ *Col2Cre*^−^ and *Col2Cre*^+^ growth plates at P21. **b** 2 h BrdU labelling of proliferation in *Manf*^fl/fl^ *Col2Cre*^−^ and *Col2Cre*^+^ growth plates at P21 showing a 29% decrease in chondrocyte proliferation upon deletion of MANF (*n* = 5). **c** TUNEL assay per zone in *Manf*^fl/fl^ *Col2Cre*^−^ and *Col2Cre*^+^ growth plates at P21 (*n* = 3). **d** Total apoptosis in *Manf*^fl/fl^ *Col2Cre*^−^ and *Col2Cre*^+^ growth plates at P21 (*n* = 3). RZ resting zone, PZ proliferative zone, HZ hypertrophic zone, NC negative control. **P < 0.01. Scale bar 200 μm

*Manf*^*fl/fl*^*Col2Cre*^+^ mice had shorter long bones at both 3 weeks (not shown) and 9 weeks of age (Fig. 2a, c; tibia ↓5.4% *P* < 0.001; femur ↓3.5% *P* < 0.001) and reduced skull lengths (measured from the tip of the nose to the base of the skull (Fig. 2a, c; skull ↓4.5% *P* < 0.05)), indicating a disruption to endochondral ossification. Interestingly, the *Manf* null cartilage growth plate appeared morphologically normal and showed the typical columnar organisation of chondrocytes that was comparable to wild-type mice (Fig. 2b; Online Resource 1, Fig. 2b). Moreover, the location of key cartilage ECM components such as types II and X collagen and matrilin-3 was unchanged in *Manf*^*fl/fl*^ *Col2Cre*^+^ growth plates, thus indicating that MANF is not essential for the processing and secretion of these key structural components of the cartilage growth plate (Fig. 3a).

### Deletion of MANF from the cartilage growth plate leads to a decrease in chondrocyte proliferation and dysregulated apoptosis

A 2-h in vivo labelling by BrdU was used to measure chondrocyte proliferation in *Manf* wild type (*Manf*^*fl/fl*^ *Col2Cre*^−^) and *Manf*^*fl/fl*^ *Col2Cre*^+^ growth plate cartilage at 3 weeks of age. We measured a 29% decrease in chondrocyte proliferation following MANF deletion suggesting an underlying cellular stress (Fig. 3b; *P* < 0.01, *n* = 5,).

The TUNEL assay was used to identify apoptosis in wild type (*Manf*^*fl/fl*^ *Col2Cre*^−^) and *Manf*^*fl/fl*^ *Col2Cre*^+^ growth plate cartilage at 3 weeks, and TUNEL positive cells were individually quantified in the resting, proliferative and hypertrophic zones respectively. We detected no differences in chondrocyte apoptosis in the resting or hypertrophic zones of *Manf*^*fl/fl*^ *Col2Cre*^+^ growth plate cartilage when compared to the wild-type (*Manf*^*fl/fl*^ *Col2Cre*^−^) littermates, but a 2.5-fold increase in TUNEL positive cells in the proliferative zone (Fig. 3c; *n* = 3). However, the overall proportion of TUNEL positive cells in the total growth plate was similar between the *Manf*^*fl/fl*^ *Col2Cre*^−^ and *Manf*^*fl/fl*^ *Col2Cre*^+^ mice, suggesting premature (stress-induced) apoptosis was occurring in chondrocytes of the proliferating zone (Fig. 3d).

### The ablation of MANF in growth plate cartilage leads to an increased ER stress response

Transcriptomic analysis (RNAseq) was performed on RNA extracted from the chondrocytes of the tibial cartilage of 5-day-old mice. Surprisingly, the expression of only seven genes was differentially changed between wild type (*Manf*^*fl/fl*^ *Col2Cre*^−^) and *Manf*^*fl/fl*^ *Col2Cre*^+^ chondrocytes (Table 1; *P* < 0.05 and a log2 fold change > 0.6).

**Table 1:** Results of the RNAseq analysis of MANF null chondrocytes at 5 days

ID	Gene name	log2 Fold change	Fold change	KOvsWT Padj
ENSMUSG00000027313	Chac1	1.0	2.0	7.00E−16
ENSMUSG00000032715	Trib3	0.7	1.6	6.63E−08
ENSMUSG00000032115	Hyou1	0.7	1.6	2.00E−18
ENSMUSG00000029752	Asns	0.7	1.6	1.23E−12
ENSMUSG00000026864	Hspa5/Grp78	0.6	1.5	7.05E−16
ENSMUSG00000022769	Sdf2l1	0.6	1.5	1.55E−06

**Fig. 4:**
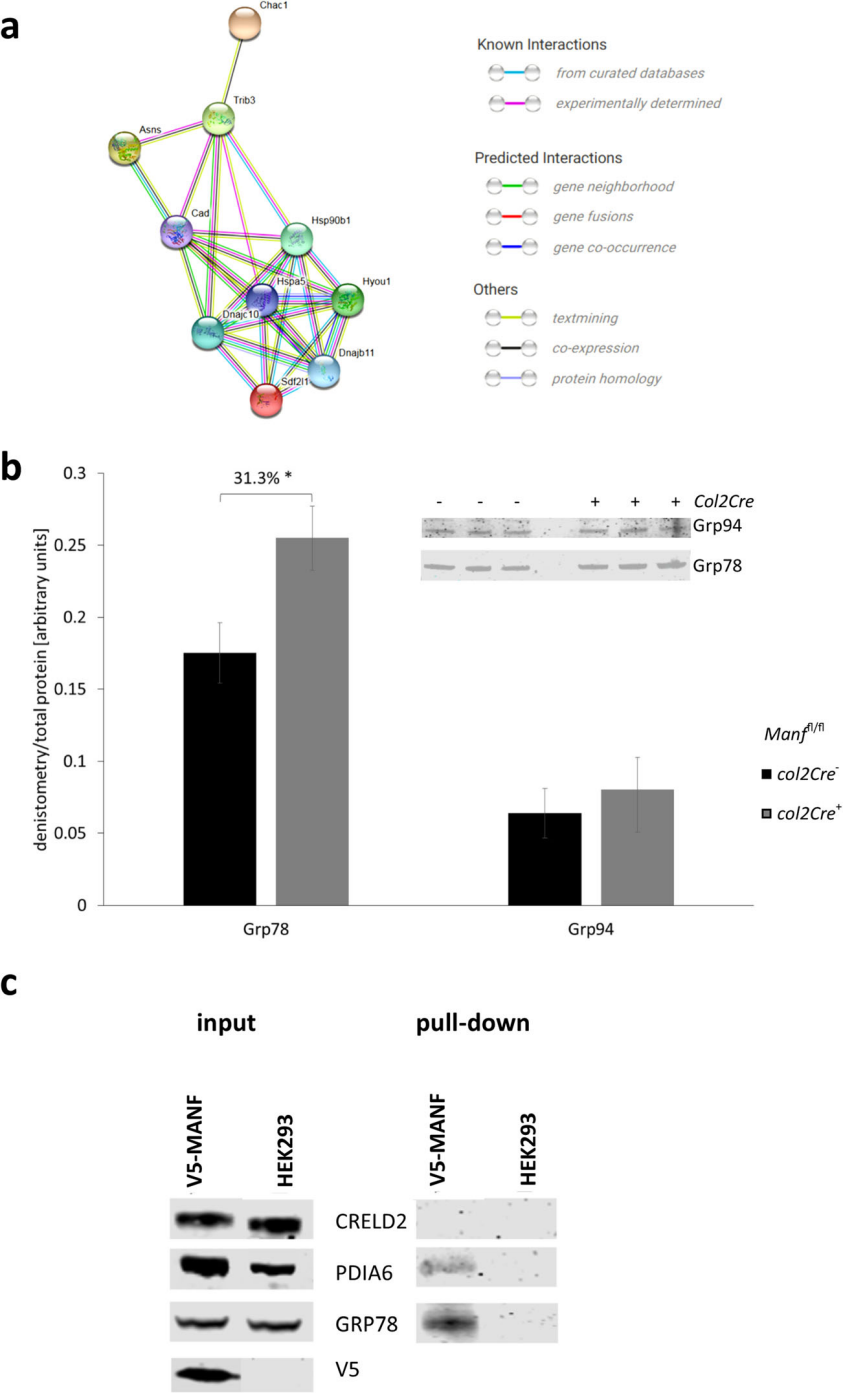
**a** STRING network showing the known interactions between the proteins encoded by the genes differentially expressed in the *Manf*^fl/fl^ *Col2Cre*^−^ and *Col2Cre*^+^ cartilage at P5. **b** Deletion of MANF increases the levels of GRP78 but not GRP94 in cartilage at P5, as shown by Western blotting and densitometry measurement (*n* = 3). **c** Western blotting of proteins co-immunoprecipitated in the 293 cells expressing FLAG-tagged recombinant MANF

The only downregulated gene was *Manf* itself, thus confirming its cartilage-specific ablation. The six upregulated genes were *Chac1* (1.9-fold *P* = 7E−16), *Trib3* (1.6-fold, *P* = 6.6E−08), *Hyou1* (1.6-fold, *P* = 2.0E−18), *Asns* (1.6-fold, *P* = 1.2E−12), *Hspa5* (1.5-fold, *P* = 7.1E−16) and *Sdf2l1* (1.5-fold, *P* = 1.6E−06). Interestingly, all these genes encode ER-resident proteins that exist as a complex inside the ER lumen, including the sentinel ER stress response marker GRP78 (*Hspa5*) (Fig. 4a). When the data were reanalysed at a less stringent 1.2-fold cutoff level, several other ER-related genes (such as *Atf5*, *Canx*, *Creld2*, *Dnajb9*, *Dnajc3*, *Edem1* and *Xbp1*; Online Resource 2) were upregulated, whilst downregulated genes pertained mostly to cell adhesion and differentiation. Moreover, *Ihh* was upregulated 1.3-fold (*P* = 0.03), potentially reflecting the dysregulated chondrocyte proliferation and differentiation. To validate the RNAseq, we confirmed that GRP78 protein (but not the related GRP94) was increased in cartilage from 5-day-old mice cartilage (Fig. 4b; 33% *P* > 0.05, *n* = 3).

### MANF directly interacts with proteins involved in the unfolded protein response

After demonstrating that a specific subset of ER stress response genes was upregulated in MANF-null chondrocytes, we next investigated if these proteins existed as a complex with MANF. Co-immunoprecipitation was performed by overexpressing FLAG-tagged MANF in 293 cells and identifying protein complexes using mass spectrometry.

MANF co-immunoprecipitated with several ER components including CCT5, GRP78, PDIA6, PRDX4 and TCP1 (Table 2; Fig. 4c; full MS analysis in Online Resource 3). MANF also bounds several ribosomal components further corroborating its role in protein synthesis and folding (Hartley et al. 2013). GO term analysis of the proteins associated with MANF clustered them into two categories: ‘protein processing in the ER’ and ‘ribosome’.

**Table 2:** Recombinant FLAG-tagged MANF pull down from whole cell lysates of transiently transfected 293 cells (spectral counting)

ID	Protein name	293 control	MANF co-IP	*P* value
ENSP00000324173	HSPA5	1	23	2.E−04
ENSP00000432799	MANF	0	5	1.E−03
ENSP00000336799	TUBA1B	0	5	2.E−03
ENSP00000280326	CCT5	0	3	7.E−03
ENSP00000272227	PDIA6	1	6	2.E−02
ENSP00000403365	PKM	0	2	3.E−02
ENSP00000393241	RPS18	0	2	3.E−02
ENSP00000317334	TCP1	0	2	3.E−02
ENSP00000368646	PRDX4	0	3	5.E−02

### Exogenous MANF rescues primary chondrocytes from tunicamycin-induced ER stress

**Fig. 5:**
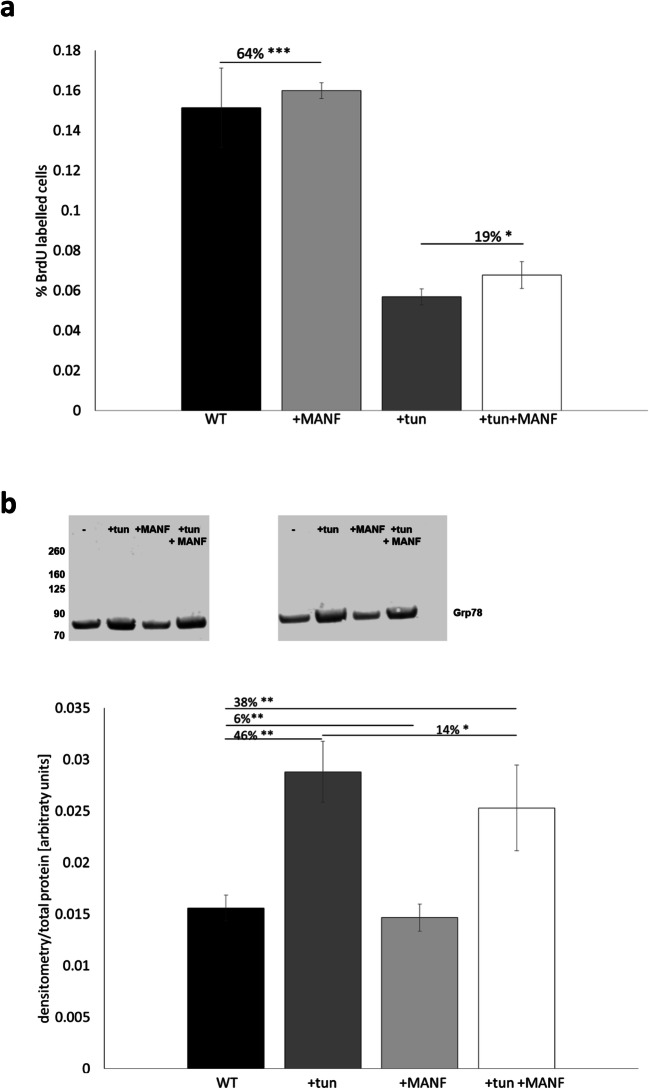
**a** BrdU labelling of control primary chondrocytes and chondrocytes treated with tunicamycin with and without exogenous MANF (*n* = 2). **b** Western blot and densitometry measurement of GRP78 levels in untreated and tunicamycin-treated primary chondrocytes with and without exogenous MANF (*n* = 2). Tun tunicamycin. ***P < 0.001, **P < 0.01, *P < 0.05

Exogenous MANF has been shown to alleviate ER stress in pancreas, brain and heart tissues (Apostolou et al. 2008; Huang et al. 2016; Zhao et al. 2013). To explore if there is a similar role for MANF in cartilage, primary chondrocytes were extracted from wild-type costochondral cartilage and treated with 1 μg/mL tunicamycin to induce ER stress by blocking *N*-linked glycosylation. The cells were labelled with BrdU for 2 h to determine the effect on cell proliferation, and not surprisingly, tunicamycin treatment significantly reduced chondrocyte proliferation (Fig. 5a: by 64%, *P* > 0.05). The addition of exogenous MANF had no significant effect on the proliferation of untreated chondrocytes; however, it led to a 19% increase in the proliferation of tunicamycin-treated chondrocytes, suggesting that it may have alleviated to some extent the tunicamycin-induced ER stress (Fig. 5a). The influence of MANF on tunicamycin-induced ER stress was further confirmed by immunoblotting for GRP78. Levels of GRP78 increased by 46% following tunicamycin treatment, indicating high levels of ER stress, but were reduced by 14% following treatment with exogenous MANF (Fig. 5b, *P* < 0.01 and *P* < 0.05, respectively). Overall, these data suggested the potential for exogenous MANF to reduce ER stress.

### MANF plays an important role in modulating the pathobiology of MED

After demonstrating that the deletion of *Manf* in mouse chondrocytes produced a growth plate dysplasia and chondrodysplasia-like phenotype, we next investigated its putative protective role in *Matn3* MED chondrocytes. *Manf*^*fl/fl*^ *Col2Cre*^+^ mice were crossed with the *Matn3*^V194D^ mouse model of MED (Leighton et al. 2007) to determine whether the upregulation of *Manf* expression in *Matn3*^V194D^ chondrocytes (Nundlall et al. 2010) was a detrimental consequence or in fact beneficial and represented an attempt by the cells to alleviate the ER stress.

**Fig. 6:**
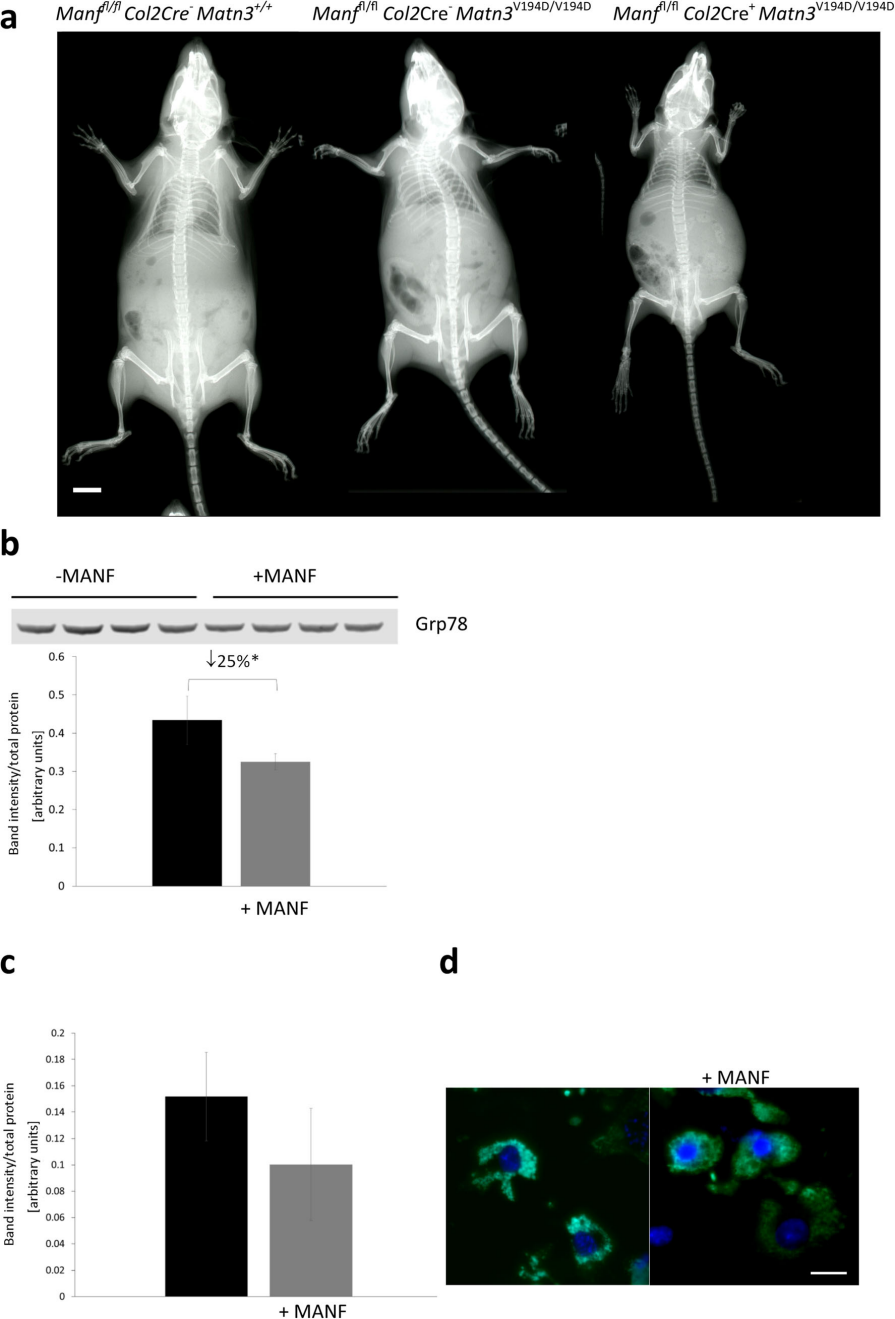
**a** X-ray radiographs of *Manf*^fl/fl^ *Col2Cre*^−^ *Matn3*^+/+^, *Manf*^fl/fl^ *Col2Cre*^−^ *Matn3*^V194D/V194D^ and *Manf*^fl/fl^ *Col2Cre*^+^ *Matn3*^V194D/V194D^ mice at 6 weeks, showing the dramatic effect of removal of MANF from cartilage of the mouse model of MED. **b** Western blot and densitometry measurement of intracellular GRP78 levels in *Matn3*^V194D/V194D^ primary chondrocytes with and without exogenous MANF (*n* = 4). **c** Western blot and densitometry measurement of intracellular matrilin-3 levels in *Matn3*^V194D/V194D^ primary chondrocytes with and without exogenous MANF (*n* = 4). **d** Immunocytochemistry for matrilin-3 showing that exogenous MANF has no effect on the intracellular retention of mutant matrilin-3 (in green) in *Matn3*^V194D/V194D^ primary chondrocytes. DAPI was used as a counterstain. Scale bars 5 mm (**a**) and 200 μm (**d**) (colour figure online)

Mice that were homozygous for both mutant alleles (*Manf*^*fl/fl*^ and *Matn3*^V194D^) and carrying a copy of *Col2Cre* were dramatically shorter than their wild-type littermates and had breathing difficulties due to restricted rib cages. This obvious increase in disease severity confirmed a chondroprotective role for MANF in the pathobiology of MED (Fig. 6a). However, in vitro treatment of *Matn3*^V194D^ chondrocytes with exogenous MANF had no effect on the amount or localisation of retained mutant matrilin-3 despite lowering the levels of GRP78 by 25% (Fig. 6b–d). Overall, these data suggested that intracellular MANF is of more importance to pathobiology of MED.

## Discussion

MANF, also known as ARMET (arginine-rich mutated in early stage tumours), is a small arginine-rich protein found in the ER (Hartley et al. 2013; Mizobuchi et al. 2007). MANF is strongly expressed in the lung epithelium, liver, pancreas and brain (Shridhar et al. 1996a, b) and is cytoprotective against ischemia-induced ER stress in several tissues (Airavaara et al. 2009; Apostolou et al. 2008; Tadimalla et al. 2008). Following ER stress, MANF can be secreted from cells due to an imperfect KDEL motif at the C-terminus (Henderson et al. 2013; Oh-Hashi et al. 2012). It has been suggested that it is through the action of cell surface KDEL receptors and a putative novel receptor that exogenous MANF exerts its cytoprotective effect (Henderson et al. 2013). MANF has a similar expression pattern to BiP (GRP78) in mouse tissues (Mizobuchi et al. 2007), and BiP inhibition blocks exogenous MANF-mediated cell survival (Huang et al. 2016). The cytoprotective effect of MANF has been shown in several tissues, but the exact mechanism remains to be elucidated with some studies showing an increase (Huang et al. 2016), whilst others report a decrease in BiP upon MANF treatment (Zhao et al. 2013).

The study of a *Manf* null mouse model (in C57BL/6 × ICR mice) has previously been published in which the mice were viable, but over time, they developed diabetes and showed signs of pancreatic ER stress (Lindahl et al. 2014). In order to study the role of MANF in cartilage health and disease, we initially tried to generate a global *Manf* knockout mouse line on a pure C57BL/6 background; however, somewhat surprisingly, hemizygous C57BL/6 *Manf*^+/−^ mice failed to produce viable *Manf* null offspring. Indeed, *Manf* null pups died perinatally through respiratory arrest due to a lung malformation characterised by decreased alveolar space and volume of individual alveoli. Interestingly, BiP (GRP78) has been shown to regulate distal epithelial cell survival during lung development and lungs from GRP78 null mice exhibit dilated alveolar airspaces and alveolar hypoplasia leading to perinatal respiratory arrest (Flodby et al. 2016). Deletion of *Manf* induces *Hspa5* expression (Lindahl et al. 2014 and in this study) and the phenotype of the *Manf* null lungs are directly opposite to the phenotype seen following deletion of *Hspa5* (Flodby et al. 2016), indicating that the GRP78/MANF relationship is important for lung tissue development.

MANF was previously identified as the most highly upregulated gene in a mouse model of MED resulting from a mutation in matrilin-3 (Nundlall et al. 2010). However, the role of MANF in cartilage and in the pathobiology of chondrodysplasias remains largely unknown. In order to study the specific effects of *Manf* ablation in cartilage, we generated a cartilage-specific knockout strain using *Cre* recombinase expressed under the type II collagen promoter (Sakai et al. 2001). The resultant conditional knockout mice had shorter bones, but the cartilage growth plate appeared morphologically normal with no overt ECM disruption. Interestingly, chondrocyte proliferation was significantly decreased in the *Manf* null cartilage when compared to the wild-type controls and apoptosis, whilst not increased overall, was dysregulated. We have previously shown that a reduction in chondrocyte proliferation is a key mechanistic finding in mouse models of chondrodysplasia caused by mutations in *Matn3* (V194D ↓16%; Leighton et al. 2007) and COMP (T585M ↓24% (Pirog-Garcia et al. 2007) and 469Del ↓17% (Suleman et al. 2011)) and confirmed that reduced proliferation is the major driver of reduced long bone growth in mouse ER stress phenocopies (*ColIITg*^*rdw*^ ↓21% (Gualeni et al. 2013) and *ColIITg*^*cog*^ ↓12% (Rajpar et al. 2009)).

Transcriptomic analysis of chondrocytes from postnatal day 5 *Manf*^*fl/fl*^ *Col2Cre*^*+*^ cartilage revealed increased expression of only six genes whose protein products are all localised to the ER. These were the following: *Chac1* (ChaC glutathione-specific gamma-glutamylcyclotransferase 1), an ER stress-related apoptosis and oxidative stress modulator downstream of ATF4 (Mungrue et al. 2009; Crawford et al. 2015); *Trib3* (Tribbles pseudokinase 3), involved in modulation of CHOP-dependent cell death during ER stress (Fang et al. 2014; Qian et al. 2008) and decrease of proliferation of bone marrow-derived mesenchymal stem cells (Zhang et al. 2017); *Hyou1* (hypoxia upregulated 1), shown to suppress hypoxia and CHOP-induced apoptosis (Wu et al. 2013; Ozawa et al. 1999); *Asns* (asparagine synthetase), an antiapoptotic molecule localised downstream of ATF4 in cancer and ER stress (Gjymishka et al. 2009; Balasubramanian et al. 2013); *Hspa5* (the gene for GRP78), an upregulation of which inhibits CHOP-mediated apoptosis (Xiong et al. 2015); and *Sdf2l1* (stromal cell-derived factor 2 like 1), an ER stress inducible gene interacting with GRP78 and modulating the activity of the ERAD system (Tiwari et al. 2013; Fujimori et al. 2017; Fukuda et al. 2001). In order to verify the RNAseq results, and to look for MANF-associated protein complexes, we performed co-immunoprecipitation by overexpressing FLAG-tagged recombinant MANF in 293 cells. Interestingly, MANF co-immunoprecipitated with GRP78, PRDX4 and PDIA6 amongst others, further confirming MANF’s involvement in ER chaperone complexes. Interestingly, all of the protein products of the genes upregulated in the absence of *Manf* are linked to the PERK arm of the UPR and modulation of ER stress-related apoptosis and have been shown to interact with each other (Zhang et al. 2002; Fels and Koumenis 2006; Kang et al. 2017; Meunier et al. 2002). Moreover, this finding is in agreement with data published for the *Manf* null pancreas, which also showed a specific upregulation of the PERK arm of the UPR pathway including GRP78, ATF4, PERK and CHOP (Lindahl et al. 2014).

Overall, these data suggest that the deletion of *Manf* introduces an imbalance in the chondrocyte ER homeostasis that in itself is sufficient to result in a chondrodysplasia-like phenotype. Chondrocytes in developing cartilage are highly secretory cells, producing a proteoglycan and a collagen-rich ECM, and exist in a state of mild physiological ER stress (Hughes et al. 2017; Hino et al. 2014). We have previously shown that inducing additional ER stress in growth plate chondrocytes, by engineering the *Col2a1* promoter–driven expression of misfolded and intracellularly retained thyroglobulin, is enough to induce a decrease in chondrocyte proliferation and resultant dwarfism independent of apoptosis (Rajpar et al. 2009; Gualeni et al. 2013). Here, we show that disrupting the chondrocyte UPR machinery, by removing a single UPR component, induces ER stress and has a comparable effect on endochondral ossification.

As previously stated, *Manf* was the highest upregulated gene detected in the chondrocytes of a mouse model of MED resulting from the aggregation of misfolded mutant matrilin-3 in the ER lumen (Leighton et al. 2007; Nundlall et al. 2010). To determine whether the upregulation of *Manf* in these ‘MED chondrocytes’ was beneficial or in itself a detrimental side effect of ER dysregulation, we crossed the *Manf*^*fl/fl*^ *Col2Cre*^+^ mice with our *Matn3*^V194D^ model of MED. Interestingly, the deletion of *Manf* from *Matn3* mutant chondrocytes resulted in an aggravation of the MED skeletal phenotype with the ‘double mutant’ mice exhibiting further shortened bones and bell-shaped rib cages that impeded their breathing. Based on this finding, we postulate that the upregulation of *Manf* expression in the MED chondrocytes has a beneficial role and may be a potential therapeutic target.

MANF can also be upregulated during tunicamycin-induced ER stress (Liu et al. 2016; Wang et al. 2015) and is secreted under ER stress due to being outcompeted for binding to the intracellular KDEL receptors (Oh-Hashi et al. 2012; Henderson et al. 2013). Exogenous MANF has been shown to have an anti-apoptotic, pro-proliferative and anti-ER stress influence in several cell types (Cunha et al. 2017; Airavaara et al. 2009; Gao et al. 2017). Here, we show a similar effect of exogenous MANF on ER-stressed chondrocytes, as evidenced by the reduced levels of GRP78 and increased cell proliferation of tunicamycin-treated primary chondrocytes following the addition of MANF. However, although the treatment of matrilin-3 mutant cells with exogenous MANF reduced the levels of GRP78, it did not reduce the levels of mutant matrilin-3 retention, indicating that it is the intracellular MANF that plays a beneficial role in the pathobiology of matrilin-3 related MED.

To conclude, these data, in conjunction with our previous studies, demonstrates that ER homeostasis is essential for cartilage development and bone growth and that ER stress provides a defined genetically tractable target for therapy.

## Electronic supplementary material

ESM 1 (PDF 2111 kb)

ESM 2 (PDF 231 kb)

ESM 3 (XLSX 27 kb)
