# Supplementary material for: Mesencephalic astrocyte-derived neurotropic factor is an important factor in chondrocyte ER homeostasis
Source: Cell Stress Chaperones. 2018 Dec 12;24(1):159–73. doi: 10.1007/s12192-018-0953-7 (PMC6363614; doi:10.1007/s12192-018-0953-7)

Mesencephalic astrocyte-derived neurotropic factor is an important factor in chondrocyte ER homeostasis.

Cell Stress and Chaperones

Bell PA[1#], Dennis EP[1,2#], Hartley CL[2*], Jackson RM[1], Porter A[3], Treumann A[3], Boot-Handford RP[2], Pirog KA[1$], Briggs MD[1,2].

[1] Institute of Genetic Medicine, Newcastle University, International Centre for Life, NE1 3BZ Newcastle Upon Tyne, UK

[2] Wellcome Trust Centre for Cell-Matrix Research, University of Manchester, Oxford Road, M13 9PT Manchester, UK

[3] Newcastle University Protein and Proteome Analysis Facility, Newcastle University, NE1 7RU Newcastle Upon Tyne, UK

[#] both authors contributed equally to this work

* current affiliation: Genomic Diagnostics Laboratory, Manchester Centre for Genomic Medicine, Manchester University Hospitals NHS Foundation Trust, Manchester, M13 9WL

[$] corresponding author: katarzyna.pirog@ncl.ac.uk +44(0) 191 241 8816

Figure 1. a) Hematoxylin and eosin staining of cross sections of *Manf*$^{+/+}$ and *Manf*$^{-/-}$ lungs at E17.5, E18.5 and E19.5 showing a decrease in alveolar space across the ages. **b)** Quantification of the alveolar number, alveolar space per section and average alveolar space at E17.5, E18.5 and E19.5 (n=2). Key: ** P<0.01, * P<0.05, # P~0.05 (approaching significance).

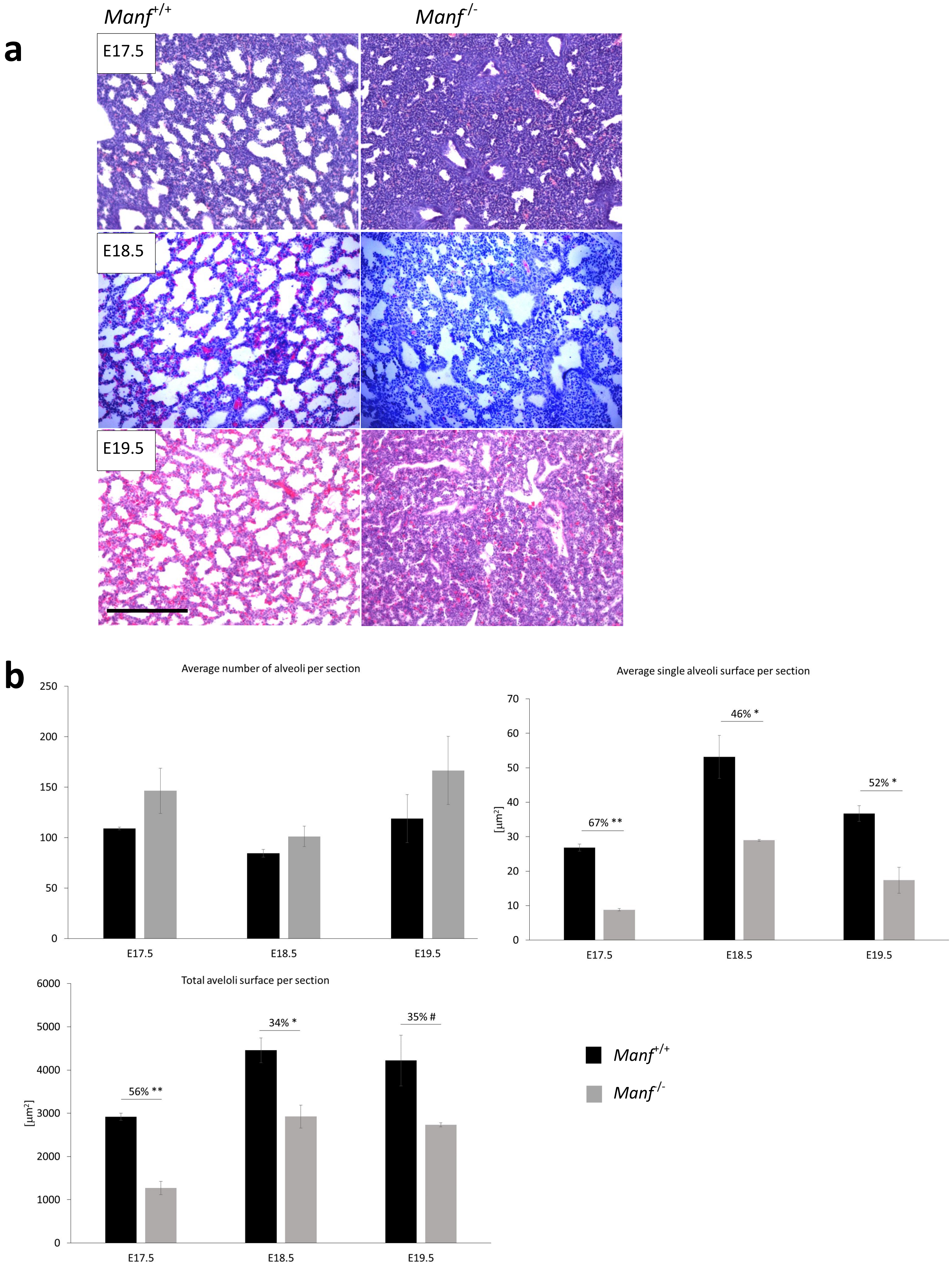

Figure 2. a) X-rays of *Manf*$^{+/+}$ and *Manf*$^{/-}$ 50% C57BL6/J 50% 129Sv mice at 3 weeks showing the effect of global MANF deletion on long bone growth. **b)** Hematoxylin and eosin staining of cartilage growth plates from *Manf*$^{+/+}$ and *Manf*$^{/-}$ 50% C57BL6/J 50% 129Sv mice at 3 weeks showing normal growth plate organisation in wild type and MANF null mice at 3 weeks. Key: scale bar = 200um.

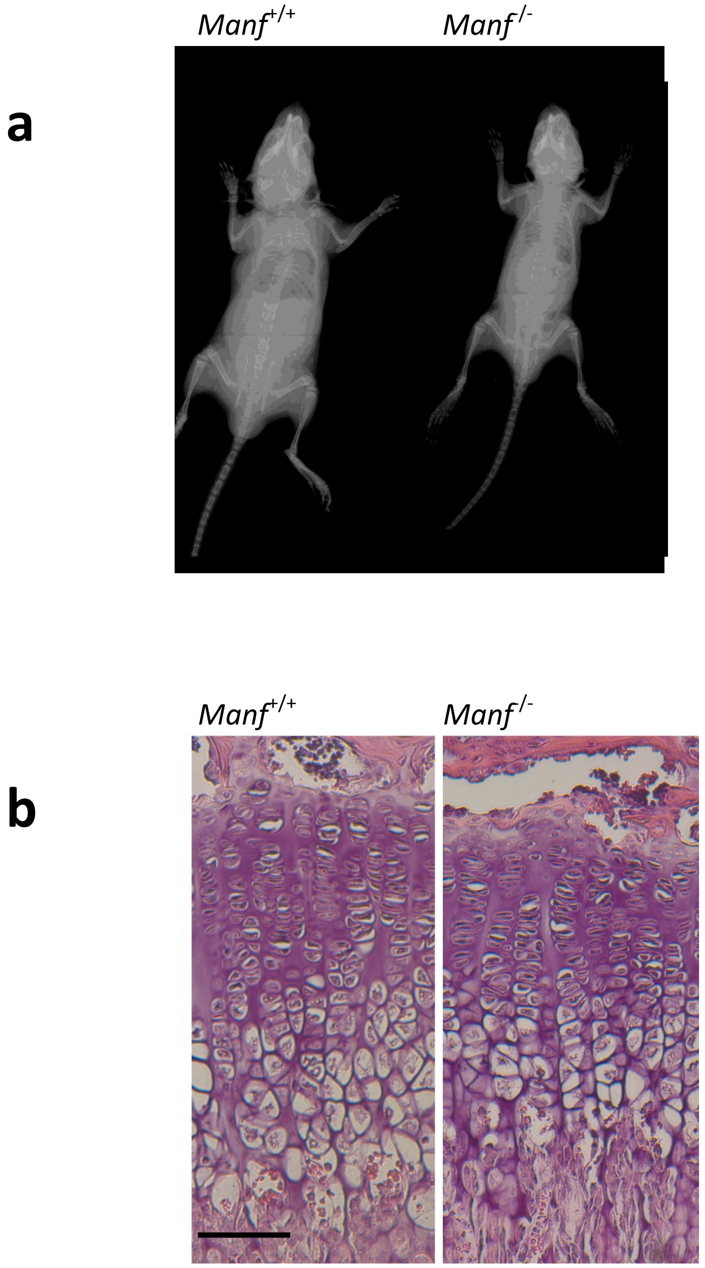

Figure 3. a) Loading control (total protein) for Figure 4b and c. **b)** Loading Loading control (total protein) for Figure 5b. **c)** Loading control for Figure 6 b and c.

a

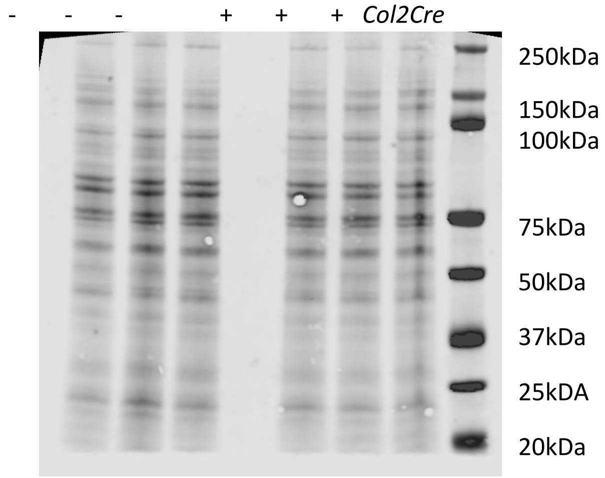

B

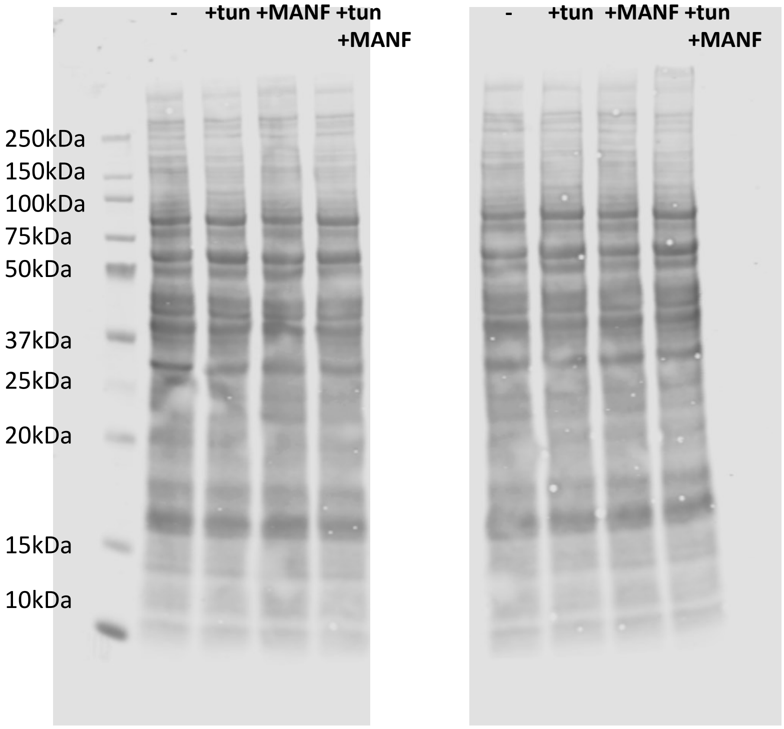

c

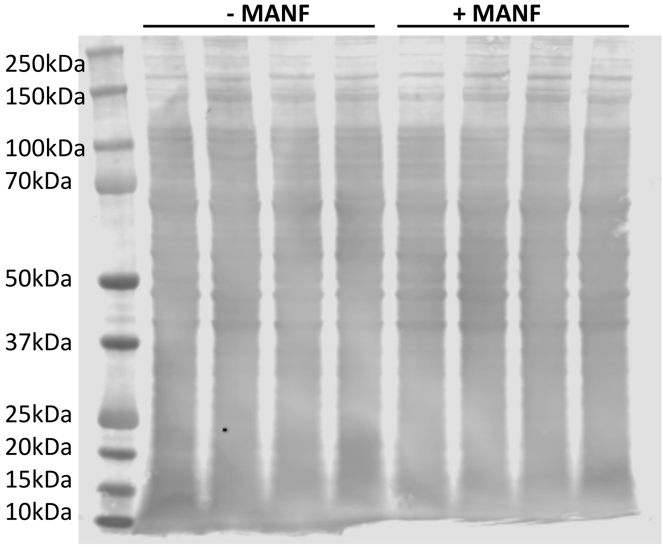

Supplement: Supplementary file 1 — (PDF 2111 kb) [file 12192_2018_953_MOESM1_ESM.pdf]
