# Supplementary material for: Mesencephalic astrocyte-derived neurotropic factor is an important factor in chondrocyte ER homeostasis
Source: Cell Stress Chaperones. 2018 Dec 12;24(1):159–73. doi: 10.1007/s12192-018-0953-7 (PMC6363614; doi:10.1007/s12192-018-0953-7)
Supplement: Supplementary file 2 — (PDF 231 kb) [file 12192_2018_953_MOESM2_ESM.pdf]

Mesencephalic astrocyte-derived neurotropic factor is an important factor in chondrocyte ER homeostasis.

Cell Stress and Chaperones

Bell PA[1#], Dennis EP[1,2#], Hartley CL[2*], Jackson RM[1], Porter A[3], Treumann A[3], Boot-Handford RP[2], Pirog KA[1$], Briggs MD[1,2].

[1] Institute of Genetic Medicine, Newcastle University, International Centre for Life, NE1 3BZ Newcastle Upon Tyne, UK

[2] Wellcome Trust Centre for Cell-Matrix Research, University of Manchester, Oxford Road, M13 9PT Manchester, UK

[3] Newcastle University Protein and Proteome Analysis Facility, Newcastle University, NE1 7RU Newcastle Upon Tyne, UK

[#] both authors contributed equally to this work

* current affiliation: Genomic Diagnostics Laboratory, Manchester Centre for Genomic Medicine, Manchester University Hospitals NHS Foundation Trust, Manchester, M13 9WL

[$] corresponding author: katarzyna.pirog@ncl.ac.uk +44(0) 191 241 8816

Table 1 A list of all statistically significantly changed genes in the RNAseq analysis of *Manf*$^{fl/fl}$ *Col2Cre*$^{+}$ vs *Manf*$^{fl/fl}$ *Col2Cre*$^{-}$ cartilage at postnatal day 5. Fold changes in log2 scale. Significantly changed genes at 1.5-fold cut-off have been highlighted in bold.

**P5 UPREGULATED GENES**				**P5 DOWNREGULATED GENES**			
**ID**	**gene name**	**KOvsWT log2 Fold-Change**	**KOvsWT _padj**	**ID**	**gene name**	**KOvsWT log2 Fold-Change**	**KOvsWT _padj**
ENSMUSG00000027313	**Chac1**	0.96684	7.00E-16	ENSMUSG00000103212	AC132393.1	-0.21312	0.020845
ENSMUSG00000032715	**Trib3**	0.69724	6.63E-08	ENSMUSG00000045573	Penk	-0.24892	0.044725
ENSMUSG00000032115	**Hyou1**	0.69158	2.00E-18	ENSMUSG00000041577	Prelp	-0.27017	0.025803
ENSMUSG00000029752	**Asns**	0.66058	1.23E-12	ENSMUSG00000034612	Chst11	-0.29682	0.003493
ENSMUSG00000026864	**Hspa5**	0.63051	7.05E-16	ENSMUSG00000003402	Prkcsh	-0.30336	0.010775
ENSMUSG00000022769	**Sdf2l1**	0.60545	1.55E-06	ENSMUSG00000043587	Pxylp1	-0.33952	0.011419
ENSMUSG00000078670	Fam174b	0.51797	0.000325	ENSMUSG00000028211	Trp53inp1	-0.34019	0.038522
ENSMUSG00000020571	Pdia6	0.50429	1.38E-12	ENSMUSG00000002732	Fkbp7	-0.36643	0.002987
ENSMUSG00000031232	Magt1	0.50325	3.66E-07	ENSMUSG00000030077	Chl1	-0.37961	0.035862
ENSMUSG00000030717	Nupr1	0.49545	3.66E-07	ENSMUSG00000064246	Chil1	-0.38275	0.029398
ENSMUSG00000020048	Hsp90b1	0.49220	1.62E-11	ENSMUSG00000033036	Gm7879	-0.38920	1.34E-05
ENSMUSG00000038539	Atf5	0.46326	5.90E-06	ENSMUSG00000029016	Clcn6	-0.39150	0.0073
ENSMUSG00000014905	Dnajb9	0.46205	0.000468	ENSMUSG00000030111	A2m	-0.39392	1.76E-07
ENSMUSG00000023272	Creld2	0.45976	5.30E-07	ENSMUSG00000039542	Ncam1	-0.39395	0.017751
ENSMUSG00000035227	Spcs2	0.45919	6.16E-07	ENSMUSG00000028619	Tceanc2	-0.40241	0.034292
ENSMUSG00000025185	Loxl4	0.45367	0.001904	ENSMUSG00000075318	Scn2a1	-0.41590	0.022361
ENSMUSG00000054408	Spcs3	0.43682	5.30E-07	ENSMUSG00000021185	9030617O03Rik	-0.42418	0.018513
ENSMUSG00000020368	Canx	0.43036	1.75E-07	ENSMUSG00000002985	Apoe	-0.43188	0.000336
ENSMUSG00000031490	Eif4ebp1	0.42313	0.000346	ENSMUSG00000028532	Cachd1	-0.43628	0.004229
ENSMUSG00000031297	Slc7a3	0.42171	0.010255	ENSMUSG00000078719	Msmp	-0.44447	0.00105
ENSMUSG00000006538	Ihh	0.41009	0.025317	ENSMUSG00000074968	Ano3	-0.44534	0.009304
ENSMUSG00000015134	Aldh1a3	0.40359	0.033793	ENSMUSG00000026173	Plcd4	-0.45800	0.006123
ENSMUSG00000082697	Gm12913	0.40117	0.033793	ENSMUSG00000028453	Fancg	-0.48859	0.001904
ENSMUSG00000020873	Slc35b1	0.40063	0.000468	ENSMUSG00000071650	Ganab	-0.53674	3.06E-13
ENSMUSG00000028648	Ndufs5	0.39699	0.012446	ENSMUSG00000081824	BC002163	-0.63268	2.54E-11
ENSMUSG00000101188	Eif4a-ps4	0.39253	0.003791	ENSMUSG00000032575	**Manf**	-0.78863	2.07E-14
